# Do Age-Friendly Community Initiatives Form a Biopolitical Paradigm?

**DOI:** 10.1093/geroni/igaa057.2391

**Published:** 2020-12-16

**Authors:** Jarmin Yeh, Pat Fox, David Vlahov, Howard Pinderhughes

**Affiliations:** 1 University of California, San Francisco, San Francisco, California, United States; 2 Yale University, Orange, Connecticut, United States

## Abstract

Biopolitical paradigms are frameworks that specify how concerns about health and the body are made the simultaneous focus of biomedicine and state policy (Foucault 1984; Rose 2007). As aging and urbanization trends converge, developing “age-friendly community initiatives” (AFCIs) has become a global movement and important policy area. To prompt critical questioning, situational analysis was used as a theory-methods package to compare AFCI conceptual frames with perspectives of thirteen AFCI experts and seventeen older San Franciscans. Preliminary analysis suggests AFCIs form a biopolitical paradigm because they not only seek to rework boundaries between bodies and environments, they operate as modes of individual and population governance for the sake of health; yet, struggle to find ways to preserve the inclusion of older people in the ongoing social system. Understanding how AFCIs place social and physical environments squarely in view within the biomedical arena of the gerontological gaze has theoretical and policy implications.

